# Sustainability outcomes and policy implications: Evaluating China’s “old urban neighborhood renewal” experiment

**DOI:** 10.1371/journal.pone.0301380

**Published:** 2024-04-30

**Authors:** Rui Wang, Hong Wu, Robert Chiles, Yizhao Yang

**Affiliations:** 1 School of Art and Design, Wuhan University of Technology, Wuhan, Hubei Province, China; 2 Department of Landscape Architecture, The Pennsylvania State University, University Park, Pennsylvania, United States of America; 3 Department of Agricultural Economics, Sociology, and Education, Department of Food Science, Rock Ethics Institute, The Pennsylvania State University, University Park, Pennsylvania, United States of America; 4 School of Planning, Public Policy, and Management, University of Oregon, Eugene, Oregon, United States of America; University of Naples Federico II: Universita degli Studi di Napoli Federico II, ITALY

## Abstract

Globally, old urban neighborhood transformation has become a new urban sustainability focus for its significant contribution to the United Nation’s Sustainable Development Goal 11. A regeneration-oriented approach is particularly important for Chinese cities with a dwindling land supply, obsoleting infrastructure, and inadequate standard of living. Using a mixed-methods approach informed by BREEAM Communities, we examined two Chinese initiatives—old urban neighborhood renewal (OUNR) and sponge city development (SCD)—through a comprehensive study of pilot project sustainability, policy emphases and gaps, and broader governance implications. We found that SCD’s top-down technocratic management was highly efficient in enhancing neighborhood hydrological functions and physical environment. However, successes were undermined by the lack of climate considerations and civic participation. Besides actionable recommendations for applied scholarship and policymaking in China, we provide insight into how the OUNR/SCD initiatives may broadly inform worldwide urban regeneration practices through project and policy experimentations that build adaptive capacity.

## 1. Introduction

For many Chinese cities with dwindling land supply and obsoleting infrastructure, regenerating urban neighborhoods has become a critical pathway for achieving urban sustainability. Many urban neighborhoods in China developed before 2000 had minimal sustainability considerations. Their regeneration must confront multifaceted issues. China’s old urban neighborhood renewal (OUNR) policies and processes, after multiple updates in the past 20 years, have expanded from simply upgrading housing stock to greening the entire neighborhood, gradually incorporating sustainability goals in the social, economic, environmental, and institutional dimensions [1]. This article engages with the increasing call for improving long-term urban sustainability by evaluating the outcomes of pilot OUNR projects under a recent urban sustainability campaign—the Sponge City Development (SCD) initiative launched in 2014 to transform cities so that they perform like sponges to manage stormwater [2].

Internationally, the convergence of neighborhood (re)development and livability traces back to the Garden City movement and neighborhood unit theory [3]. Practices have come a long way to adopt a sustainable approach. Since the 1990s, the tendencies have shifted from implementing large-scale redevelopment schemes to encouraging regeneration based on a shared vision produced from participatory processes [4]. Previous focus on slum clearance is now replaced with building sustainable neighborhoods with multiple objectives, such as strengthening the local economy, providing affordable housing, and upgrading infrastructure [5]. These efforts have been launched at various scales, ranging from street-level retrofitting (e.g., green streets in Portland, OR, USA) to eco-district redevelopment from brownfields (e.g., Hammarby, Sweden). While the forces driving these changes come from various sources, such as enlightened civil rights discourse and growing legal requirements, a common belief now lies in the mainstream approach in most democratic societies: empowering local stakeholders to manage private-public redevelopment processes is not only morally imperative but also pragmatically necessary [6].

Nonetheless, present-day neighborhood renewal grapples with critical conceptual and practical challenges, exhibiting varied manifestations across different countries shaped by distinct political-economic contexts and regeneration phases. Shared challenges encompass realizing environmental sustainability goals (e.g., renewable energy integration and climate resiliency) [7], empowering local residents, ensuring equity and social inclusion [8, 9], navigating governance barriers, and securing adequate financing and resources [10]. In Asian developing nations, in particular, urban renewal efforts face additional hurdles, including balancing economic growth with environmental conservation [11, 12], ensuring equitable development in rapid urbanization [9], protecting cultural heritage against modernization pressures [13], ensuring adequate infrastructure and service delivery, and addressing issues such as corruption, bureaucratic inefficiencies, and limited financial resources [14].

In mainland China, neighborhood renewal practices have been led by the state, following, to a great extent, priorities identified in the national development agenda. The recent emphasis on ecological restoration as part of neighborhood redevelopment, for example, responds to the country’s shift toward a more environmentally sensitive approach [15]. Operating within China’s top-down planning and policy framework, local governments tend to align their strategies with national policy initiatives. Indeed, many recent OUNR projects are connected to a parallel national environmental initiative—the Sponge City Development mentioned above. The convergence of SCD and OUNR since 2015 has led to over 2,576 “old urban neighborhood sponge transformation” projects nationwide by 2020 [16]. While this convergence has expanded OUNR’s scope and sustainability goals, the pilot projects’ sustainability performance remains to be assessed.

Comprehensively evaluating these projects will be timely and valuable to shed light on broader practice and policy implications. The Chinese planning tradition of using local experimentation to inform higher-level policies means that experiences gained from pilot projects will likely be scaled up to become national standards [17]. Therefore, we selected 13 redevelopment projects from four pilot sponge cities representing the best renewal efforts at the time of their construction for sustainability performance assessment. Additionally, we analyzed six of the latest national and provincial OUNR policies to identify how project evaluations could inform policy revisions. Beyond providing actionable recommendations for applied scholarship and policymaking in China, these project and policy evaluations yield insights into how Chinese renewal initiatives may broadly inform worldwide neighborhood regeneration practices. Specifically, understanding how China copes with the massive scale and rapid pace of neighborhood regeneration and how these initiatives are designed and implemented under a top-down approach while striving for social equity and inclusion can inform discussions on effective renewal strategies, financing models, and ways to engage the public under diverse governance structures.

We begin this article with an overview of the history of China’s OUNR program, neighborhood renewal practices in similar Asian countries, and general neighborhood sustainability assessment approaches. We then detail the mixed methods of integrating site investigation and resident and expert interviews based on an evaluative framework called BREEAM Communities (BREEAM-C) [18]. Following the results of project sustainability outcomes and policy assessments, we discuss critical research and practice implications for future neighborhood regeneration in China and the broader world.

## 2. Background

### 2.1 OUNR history in China

**Fig 1:**
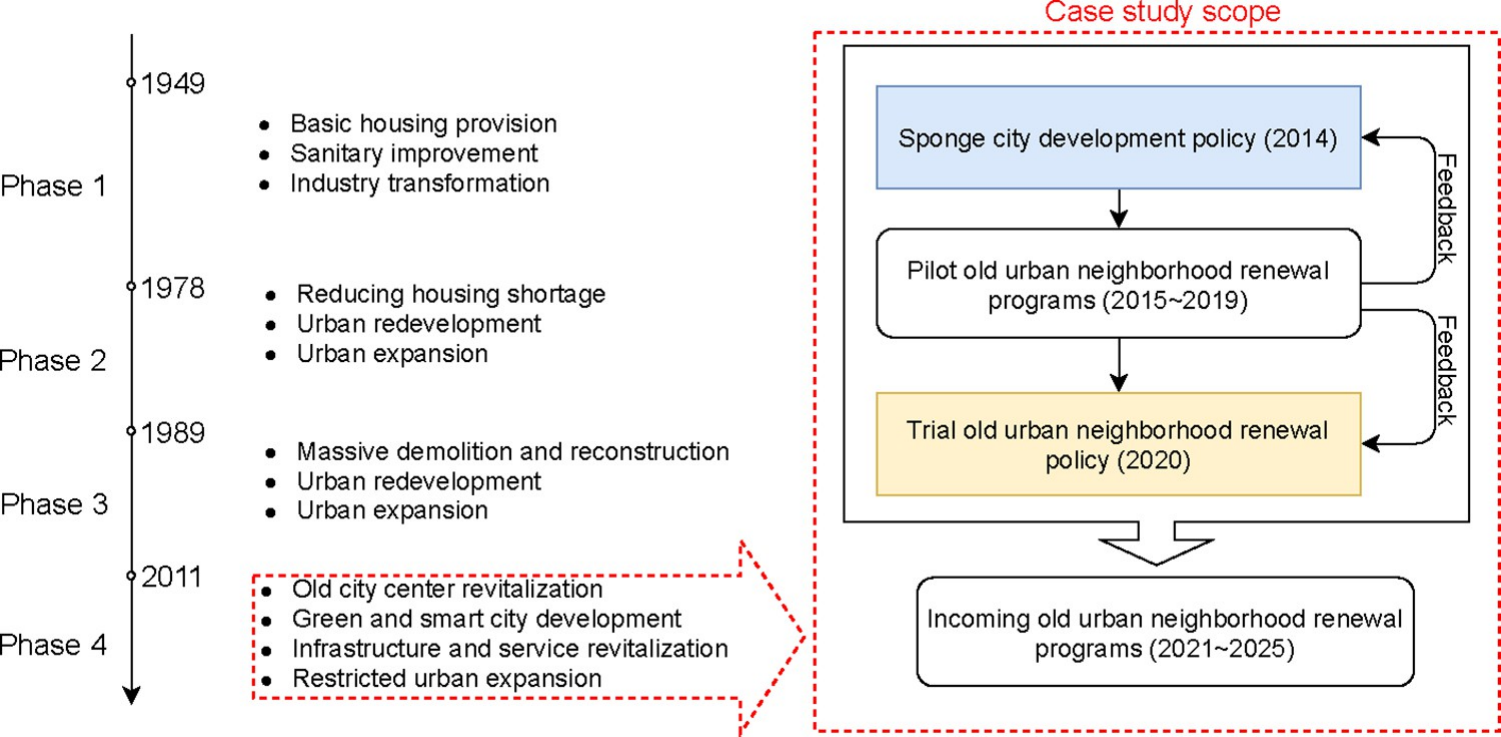
China’s urban development phases and interaction of sponge city development and old urban neighborhood renewal programs in Phase 4.

Modern China’s urban development programs have evolved over four phases [19] (Fig 1). The current Phase 4 (2011-present) reflects the national initiative to align economic and ecological goals, shifting to a more balanced approach to economic growth, restoration, and conservation [15]. Here, large-scale urban expansion and wholesale demolition and reconstruction featured in Phases 2 and 3 [20] have been gradually replaced by restoration and revitalization of old districts, neighborhoods, streets, and buildings. The overarching purpose has been to improve urban life quality, land use integration, and ecological integrity.

As mentioned above, a key linchpin of China’s top-down environmental governance has been the use of local experimentation to test different options for higher-level policies–an approach employed since the Reform and Open Era [21]. Recently, a sense of urgency to address urban sustainability has accelerated policy experiments in many urban renewal initiatives, such as the Eco-City, Sponge City, and Zero-Waste City [22]. The evolution of both the SCD and OUNR programs reflects this experimental strategy [1]. Thus far, the SCD initiative has allowed 30 pilot cities to explore solutions for socio-environmental issues (water in particular) under SCD regulations and technical guidelines. Influenced by SCD, the neighborhood sponge transformation projects have emphasized the adoption of low impact development (LID) practices [23], such as rain gardens and pervious pavements, to provide multiple hydrological, aesthetic, health, and recreational benefits [24]. In 2020, the OUNR program emerged at the forefront of urban policymaking, with a flux of national, provincial, and municipal policy releases, many of which present distinct influences from the SCD program. In practice, approximately 167 thousand neighborhoods underwent regeneration between 2018 and 2022, impacting around 80 million residents [25].

In theory, the local-central relationship built into the experimental policymaking process would support effective and sound policy formulation. However, thorough understandings of experimental results are often hindered by the speed of implementation, focus on immediate outcomes, and inadequate consideration of socio-environmental processes. These are especially relevant to the relatively new sponge city concept in great need of locally oriented material manifestation and operationalization. Accordingly, these challenges create a critical need to assess the pilot projects’ sustainability outcomes and policy implications.

### 2.2. Neighborhood regeneration in Asian developing countries

In Asian developing countries, such as Malaysia and India, which share similarities with China in their primarily top-down planning paradigms, significant policies and initiatives have been launched in the last two decades to foster sustainable neighborhood (re)development. In Malaysia, the late 1990s witnessed the introduction of many planning plans, from national to local levels, aimed at rectifying the social, environmental, and economic challenges spurred by massive urban growth in the 1980s [26]. The Malaysian Green Township evaluative system emerged in the early 2010s, encouraging townships and surrounding communities to pursue a multitude of sustainability goals, from environmental conservation to improving community well-being [27]. While new and regeneration projects have certainly made achievements in areas such as accessibility, livability, and appearance [28, 29], research has also unveiled concerns, including growing class disparities, gentrification, and lack of civic engagement due to environmental injustices ingrained within the urban development process [26, 30, 31].

In India, after phases of slum upgrading (1970s–1980s) and rapid urbanization during the Liberalization Era (1990s), contemporary urban transformation initiatives have been launched periodically by the Government of India, emphasizing smart city development, urban revitalization, and heritage development [12], primarily at the city, instead of neighborhood scale. Significant initiatives, such as the Smart Cities Mission to transform 100 urban centers into smart cities [32] and the Heritage City Development and Augmentation Yojana [33] launched in the mid-2010s, aim to improve infrastructure, services, cultural identity, and livability. However, the proliferation of urban development programs also drew criticism for missing a nationwide strategic framework and proper outcome assessments. Limited assessments have shown that, for example, the Smart Cities Mission over-emphasized advancing digital technologies while neglecting environmental conservation and social infrastructure improvement for education and health equity [32, 34].

### 2.3. Neighborhood sustainability assessment

Globally, many studies have employed Neighborhood Sustainability Assessment tools to evaluate neighborhood (re)development in differing socio-economic contexts. Over 20 third-party assessment tools have been created since the 2000s [35]. These tools generally employ a comprehensive set of indicators encompassing the four aspects of environmental, social, economic, and institutional sustainability [36]. Among them, the US’s LEED-ND [37], the UK’s BREEAM-C [18], and Japan’s CASBEE-UD [38] are the three most broadly applied international tools [39] (Table 1). Despite the growing adoption of assessment tools in Asian countries, such as Malaysia’s Green Township Index [27], India’s IGBC Green Township [40], and Singapore’s Green Mark for Districts tools [41], China has not yet developed a third-party tool, except for the BEAM Plus Neighbourhood in Hong Kong primarily used in new developments [42].

**Table 1:** The three most broadly applied international tools for neighborhood sustainability assessment.

Tool	Institution	Major themes
**LEED-ND**	US Green Building Council (USGBC)	Smart location and linkage, neighborhood pattern and design, green infrastructure and buildings, innovation and design process, regional priority credits
**BREEAM-C**	UK Building Research Establishment (BRE Group)	Governance, social and economic well-being, resource and energy, land use and ecology, and transport and movement
**CASBEE-UD**	Japan Sustainable Building Consortium (JSBC)	Resource, nature (greenery and biodiversity), artifact (building), impartiality/fairness, safety/security, amenity, traffic/urban structure, growth potential, efficiency/rationality, CO_2_ emissions from traffic sector, CO_2_ emissions from building sector, CO_2_ absorption in green sector

Regarding the assessments’ focuses and results, several European studies evaluated eco-district (re)developments and found a heavy emphasis on land use, public space, mobility, and metabolism themes, while social cohesion, economic aspects, and method and process were undervalued [43, 44]. Other US-based studies suggested underachievement in social equity and livability, such as affordable housing, crime reduction, demographic diversity, and green space provision [45, 46]. In the Asia context, several studies assessed the outcomes of pilot projects certified by Malaysia’s Green Township Index, showing deficiencies in areas such as biodiversity considerations, flood protection, low-impact materials, demographic diversity, universal accessibility [47, 48], security, and community participation [28, 29]. Neighborhood-level discourses in India mainly explored how the degree of land-use mix influenced neighborhood sustainability, with results generally supporting a moderate level of land-use mix based on travel behavior measures and resident perception [49]. Overall, the global proliferation of neighborhood sustainability assessment tools reflects their growing appeal as a policy instrument to advance neighborhood sustainability [36].

In China, because most sustainable development research and action have occurred at national to city scales, neighborhood-level research remains scarce [1, 50]. Comprehensive sustainability evaluations of the SCD/OUNR pilot projects have yet to occur. However, existing work has flagged several causes for concern: Wang et al. [51] found a low level of perceived environmental benefits by residents; Gong et al. [52] reported limited resident involvement in the renewal process; and Gu et al. [53] found low resident satisfaction towards sponge facility maintenance, hydrological performance, and public participation. The need to assess pilot projects of urban neighborhood transformation is evident.

With the above background, knowledge gaps, and assessment tool in mind, we address the following two specific questions:

RQ1 (Sustainable development outcomes): To what extent did pilot old neighborhood sponge transformation projects achieve environmental, social, economic, and institutional sustainability?
RQ2 (Policy implications): To what extent have the lessons and experiences gained from pilot projects been reflected in the latest neighborhood renewal policies? What broader implications can be drawn from the project and policy experiments?

## 3. Methods

**Fig 2:**
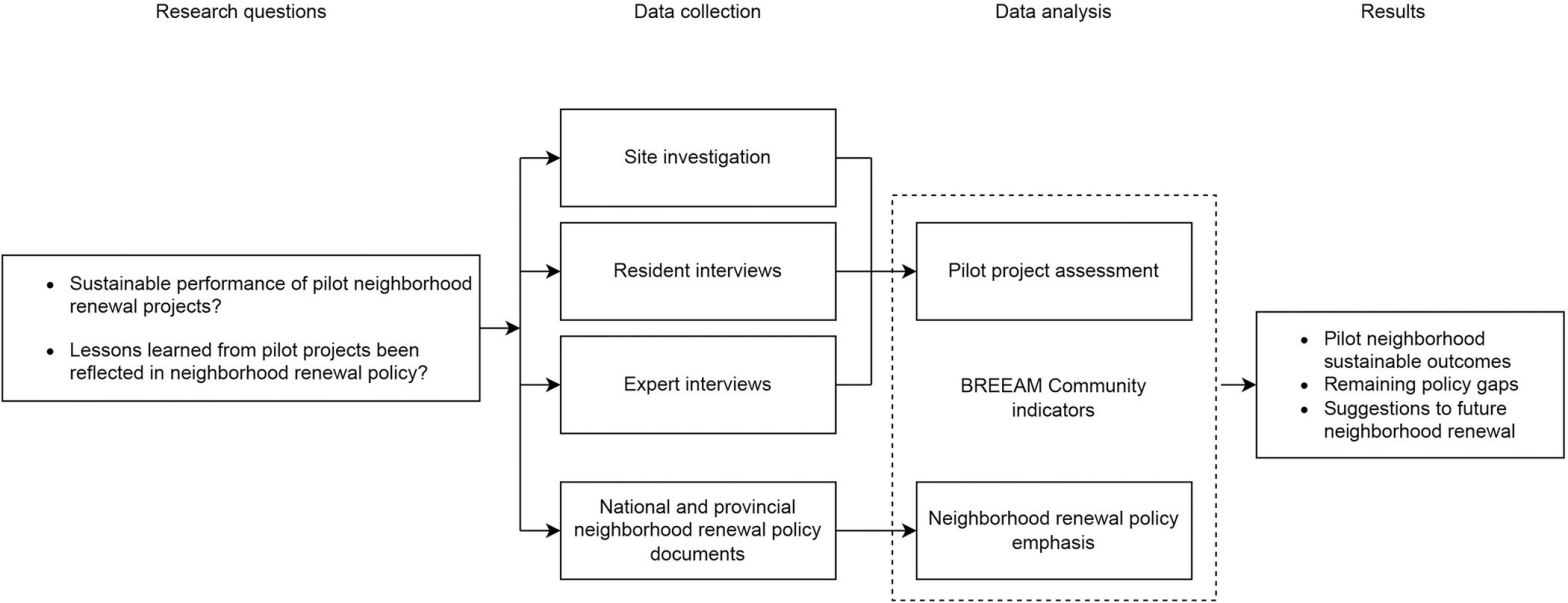
Mixed-methods research design.

We used a mixed-methods research design (Fig 2) encompassing three components: site investigations, resident and expert interviews, and policy document analysis. The site investigations and interviews address the first research question of evaluating pilot project sustainable performance. More specifically, site investigations allowed examination of the neighborhoods’ physical changes from the sponge transformation, while the resident and expert interviews helped gain insights into the benefits and challenges of the transformation from two contrasting perspectives. The third component, policy document analysis, synthesized the emphases and gaps of recent OUNR policies and helped address the second research question of project feedback for policymaking. Organized under a common framework of BREEAM-C, these three data collection and analysis methods synergize to form a comprehensive qualitative study that connects project performance to policy implications. The research was reviewed and approved by the Institutional Review Board at The Pennsylvania State University [STUDY00013257].

Among the three globally recognized neighborhood assessment tools outlined in Table 1, i.e., LEED-ND, BREEAM-C, and CASBEE-UD, we selected BREEAM-C to analyze data from all three components. As a commercial tool originated from UK, BREEAM-C features 40 indicators (see each indicator’s definition in S1 Table) embedded within five standard assessment categories, i.e., Governance, Social and economic well-being, Resource and energy, Land use and ecology, and Transport and movement. BREEAM-C distinguished itself from the other two tools for three primary reasons. First, it has the best coverage of sustainability concerns [54] yet the fewest indicators (40 vs. 53/80 in LEED-ND and CASBEE-UD, respectively). The equal three-point scale for all criteria also allows direct comparisons across indicators and themes. Second, BREEAM-C applies to both new and redevelopment projects, whereas LEED-ND only certifies projects near completion or less than three years old. Third, despite high global recognition and market appeal, BREEAM-C has rarely been applied in China [55]. Testing it here will inform its adaptability and potential enhancement for the specific context of Chinese urban neighborhood regeneration.

Below, we elaborate on the procedure for site investigation, resident and expert interviews, and policy analysis.

### 3.1. Pilot project assessment

#### 3.1.1. Site investigations

**Fig 3:**
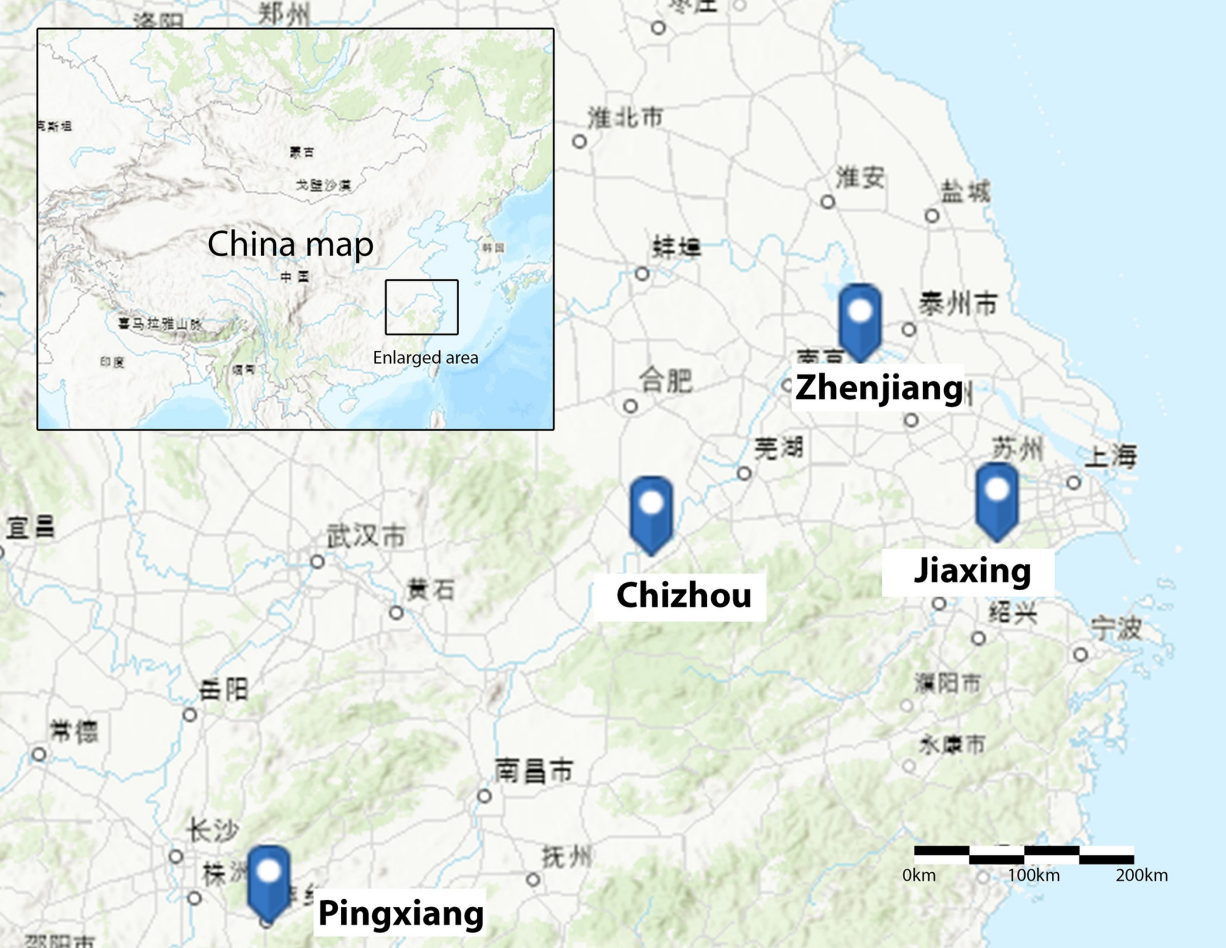
Locations of the four pilot sponge cities (a) and examples of neighborhood sponge transformation (b and c). Sources: base map of (a)—U.S. Geological Survey https://index.nationalmap.gov/arcgis/rest/services/USTopoAvailability/MapServer; public domain; (b) and (c) taken by authors.

We first employed site investigations [56] to examine physical changes from the sponge transformation. The 13 projects are from four small- to medium-sized pilot sponge cities in southeastern China, including Zhenjiang, Jiaxing, Chizhou, and Pingxiang (Fig 3, Table 2). They were selected based on scholarly literature, reports, and expert recommendations. Site investigations occurred in May 2018 and October-November 2019 by Authors 1 and 2. We focused on design, construction quality, and human interactions with the new neighborhood elements. A total of 386 photographs were taken to document visible changes (e.g., building retrofits and LIDs) and potential design, implementation, and maintenance issues. Additionally, secondary information (e.g., website news, design plans, scholarly literature, and official reports) was collected to inform previous site conditions.

**Table 2:** Background of pilot neighborhoods.

No.	Province	City	Pilot neighborhoods	Area (ha)	Year First Constructed	Year of Retrofit	No. of households	Density (# of dwellings/ha)	Average price (USD/m^2^)
**1**	Zhejiang	Jiaxing	Jueyuanchang residence	3.2	1970	2016	318	99	2157
**2**			Yanyu residence	15.1	2000	2016	2051	136	2591
**3**			Funan Garden Phase 1	9.6	2003	2016	1222	127	1864
**4**			Funan Garden Phase 2	11.7	2004	2016	1483	127	2043
**5**			Funan Garden Phase 3	9.0	2005	2015	1234	137	2088
**6**	Jiangsu	Zhenjiang	Huarun Village	3.6	1996	2016	1123	312	1421
**7**			Chashan residence	3.9	1998	2016	844	216	1562
**8**			Runjiang residence	3.0	1998	2016	802	267	1582
**9**			Jiangbin Village	11.3	1988	2015	3802	336	1082
**10**			Huashan Village Phase 1	5.5	1998	2015	1201	218	1735
**11**			Zhiye New Village	2.4	2000	2015	456	190	2108
**12**	Anhui	Chizhou	Huijing Garden Phase 1	10.9	2002	2016	1560	143	1853
**13**	Jiangxi	Pingxiang	Jindian residence	13.5	2005	2017	1127	83	844

Note: number of households and average price came from https://www.anjuke.com/; currency exchange rate based on 2021 average value: 1 USD = 6.452 CNY.

Authors 1 and 2 then applied BREEAM-C to rate 37 sustainability indicators. The remaining three, i.e., light and noise pollution and transport assessment, were not assessed (marked as “NA”) due to insufficient information. A specific indicator was scored as “1” (otherwise, “0”) when over half of the neighborhoods presented positive outcomes based on the consensus of the two raters.

#### 3.1.2. Interviews

To capture the benefits and challenges of sponge transformation, we conducted resident and expert interviews in October 2019 and May 2020. The resident interviews occurred during site visits. Twelve adults living in the studied neighborhoods before 2015 (SCD starting year) were randomly selected for face-to-face conversations lasting 10–20 minutes. The interviewees included six females and six males, eight of whom were over 60 years old. They were not asked to rate BREEAM-C indicators directly due to lack of expertise and information on many indicators (e.g., transport carbon emissions). Instead, we engaged them in informal conversations with straightforward, open-ended questions regarding their experience during and after the sponge transformation.

Areas of resident satisfaction and dissatisfaction and their rationale were first summarized using discourse analysis [57]. Authors 1 and 2 then collaboratively assessed the neighborhoods ‘ performance on the BREEAM-C indicators based on residents’ overall satisfaction and dissatisfaction. Indicators not mentioned by the residents were marked as “NM.” Because we worked with a non-representative sample, the resident interviews provided an enriched perspective instead of generalizable conclusions on resident experiences in the chosen residences. Therefore, we noted areas of satisfaction and dissatisfaction with “green” and “red” flags, respectively, to facilitate subsequent discussions. Future research with a larger sample size and probability sampling strategies is warranted to assess the generalizability of the resident perspective presented later.

For the expert interviews, we used the snowball sampling technique [58] to recruit 11 experts actively involved in OUNR/SCD research and practice. They included 4 top local government officials, 3 local SCD project managers, 2 local university professors, and 2 nationally renowned SCD designers. Here, semi-structured face-to-face or phone interviews lasting 30–90 minutes were conducted. Interviewees primarily addressed their challenges in designing, implementing, and maintaining OUNR/SCD projects. The transcripts were analyzed using discourse analysis.

The experts were also presented with the complete list and definitions of BREEAM-C indicators and asked to rate each indicator based on their overall assessment of OUNR/SCD projects in the four cities. A six-point scale was used: I = irrelevant, II = not considered, III = poor performance, IV = approaching satisfactory performance, V = satisfactory performance, and VI = strong performance. Indicators with average ratings exceeding satisfactory performance (V) received a score of “1” (otherwise, “0”).

Finally, we used the two scores of site investigation and expert assessment, along with residents’ input, to gauge the sustainability achievement for each indicator. Indicators earning an aggregated score of “2” generally represented areas of high achievement. Within this group, those red-flagged by the residents were noted and discussed for broader implications. Conversely, indicators earning an aggregated score of “0” represented areas needing the most improvement.

### 3.2. Policy assessment

To understand the emphases and potential gaps of recent OUNR policymaking, we analyzed six policy documents, one national and five provincial (Table 3), released in 2020 using qualitative data analysis software Nvivo. These policy documents were selected following a thorough review of policies from national, provincial, and municipal government websites. City-level policies were excluded due to substantial overlap with provincial policies. Both directed and summative analysis approaches [59] were employed for policy assessment. Through iterative readings, authors 1 and 2 collaboratively coded all six policy documents using the 40 BREEAM-C indicators as pre-determined codes. A phrase or sentence can be associated with one or multiple codes if it prominently conveys the concept of the respective code(s). Policy emphasis of a specific document was measured by *prevalence* [60], a direct Nvivo output defined as the percentage of text in source content associated with the respective code.

**Table 3:** Analyzed policy documents.

No.	Level		Policy type	Title	Issuing agency	Issuing time	Length (words)
**1**	National		General	National OUNR Guidance [61]	General Office of the State Council of the People’s Republic of China	2020.07	5,251
**2**	Provincial	Jiangsu	General	Guidance on OUNR Implementation [62]	Department of Housing and Urban-Rural Development, Jiangsu Province	2020.12	6,094
**3**	Provincial	Zhejiang	General	Guidance on OUNR Implementation [63]	General Office of the People’s Government of Zhejiang Province	2020.12	5,080
**4**	Provincial	Zhejiang	Technical guidance	OUNR Technical Guidance (Trial) [64]	Department of Housing and Urban-Rural Development, Zhejiang Province	2020.04	15,157
**5**	Provincial	Jiangxi	Technical guidance	OUNR Technical Guidance (Trial) [65]	Department of Housing and Urban-Rural Development, Jiangxi Province	2020.03	14,093
**6**	Provincial	Anhui	Technical guidance	OUNR Technical Guidance (Trial) [66]	Department of Housing and Urban-Rural Development, Anhui Province	2020.04	10,366

## 4. Results

**Fig 4:**
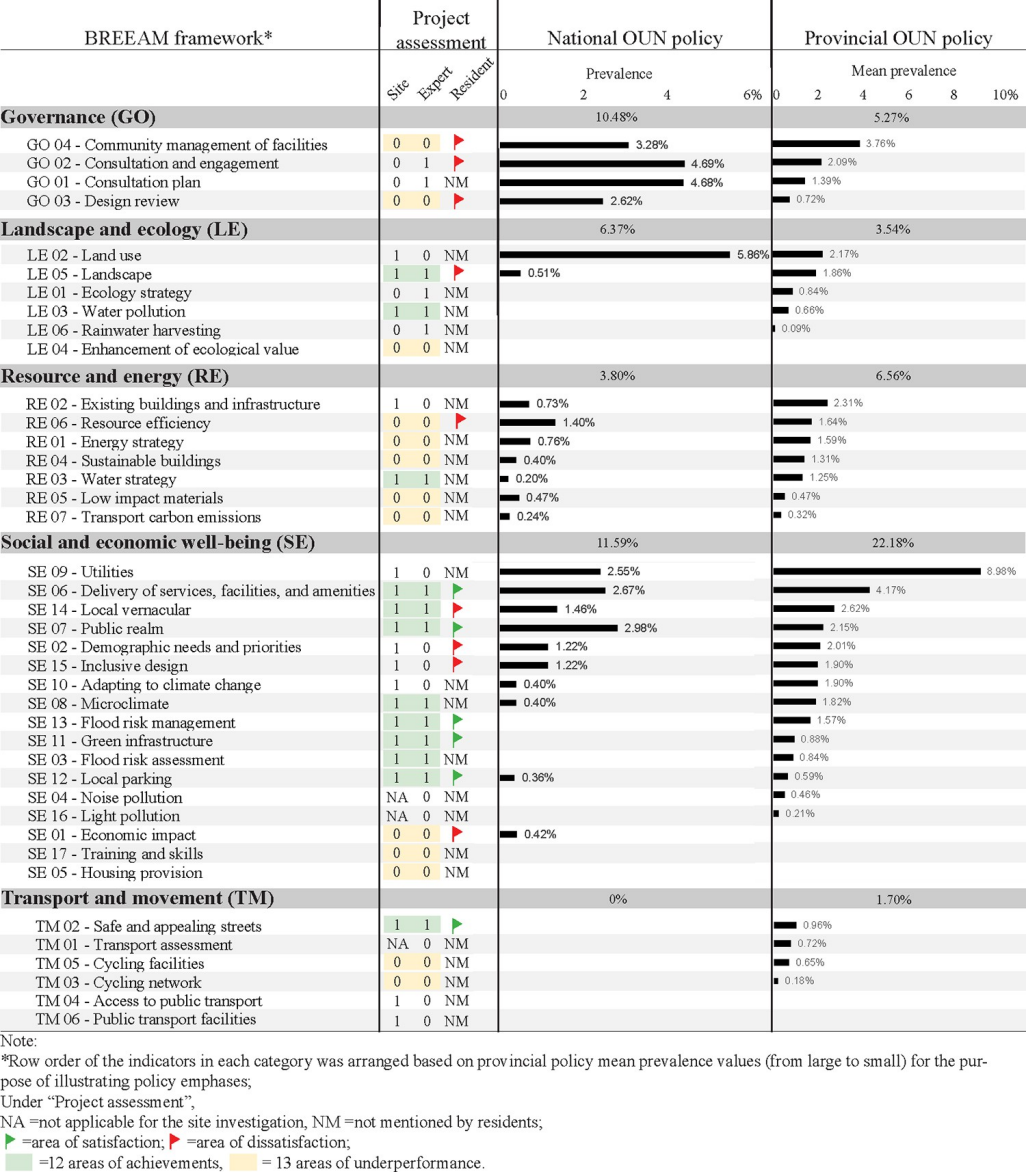
Results of pilot project assessments and policy analyses.

**Fig 5:**
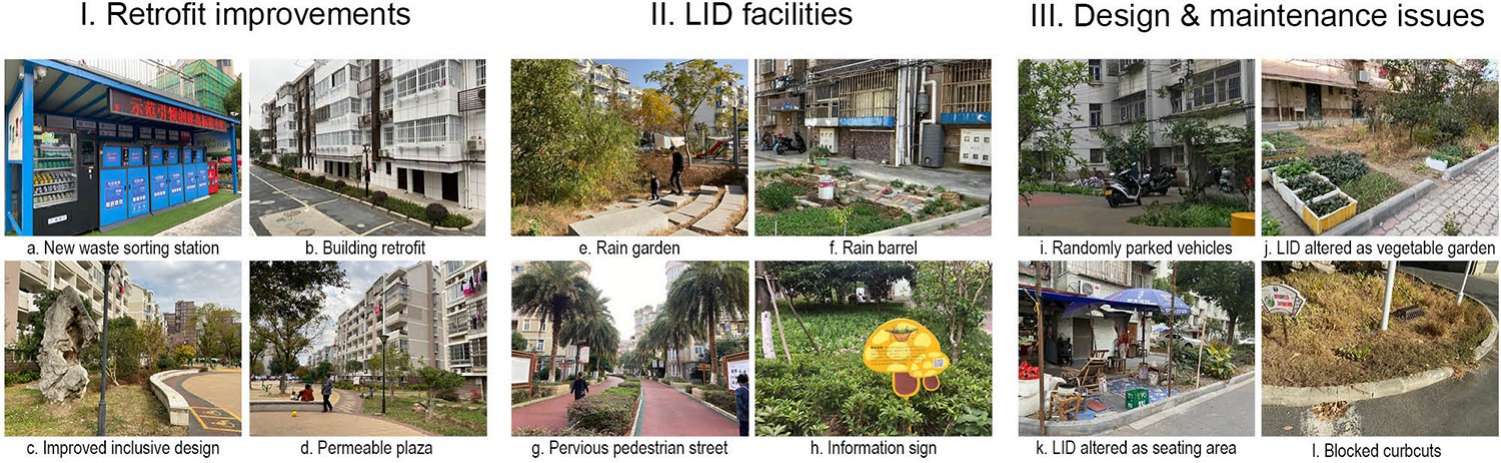
Examples of retrofit improvements, LID facilities, and design and maintenance issues. All photos by Authors 1 and 2.

The results of pilot project performances from all three perspectives and policy analyses are presented in Fig 4. Supporting images illustrating visible environment changes, LIDs implemented, and design and maintenance issues are presented in Fig 5. In what follows, we first elaborate on findings of each assessment and then synthesize how project outcomes relate to policy emphases and gaps.

### 4.1. Neighborhood sustainability outcomes

#### 4.1.1. Site assessment

Upon comparing primary and secondary information, we found that the old urban neighborhood sponge transformation was realized primarily through retrofitting shared outdoor spaces and upgrading built infrastructure. Before the renewal, for example, roads in the selected neighborhoods were predominately impervious. Green spaces drained toward paved surfaces. Roof runoff directly entered municipal sewers without being stored or infiltrated. Pedestrians and automobiles often shared roads, and parked cars occupied roadways, green spaces, plazas, and sidewalks due to severe parking shortages within and surrounding the neighborhoods. The renewal implemented both functional and cosmetic improvements (Fig 5-I). Specifically, hydrological enhancement was achieved by implementing eight types of LID facilities (Fig 5-II and S2 Table). Almost all 13 neighborhoods adopted pervious pavement, tree trenches/planters, and sunken green spaces. Rain gardens/bioswales, downspout disconnection, and educational boards were also widely used (6–9 neighborhoods), but rain barrels and green roofs were only occasionally implemented (1 neighborhood).

Among the 37 BREEAM-C indicators, 20 received a rating of “1”, indicating overall positive outcomes. They included 12 from the Social and economic well-being (SE) category, 3 each from Land use and ecology (LE) and Transport and movement (TM), and 2 from Resource and energy (RE) (see detailed rationale for each indicator in S1 Table). For the SE category, residents’ active use of shared outdoor spaces, in addition to frequent new LID installations, pavilions, playgrounds, seating and lighting, fitness equipment, and street signs, demonstrated the efficacy of Delivery of services, facilities, and amenities (SE06), Local vernacular (SE14), Public realm (SE07), Demographic needs and priorities (SE02), and Inclusive design (SE15) (Fig 4). Widespread retrofitting of downspouts and stormwater/sewage pipes led to a positive rating of Utilities (SE09). Additionally, new LIDs contributed to positive outcomes of Adapting to climate change (SE10), Microclimate (SE08), Flood risk assessment and management (SE03/13), Green infrastructure (SE11), and Local parking (SE12). For LE, retrofitting (instead of urban expansion) and the more functional and visually appealing landscapes led to positive outcomes of Land use (LE02), Landscape (LE05), and Water pollution (LE03). For RE, preserving existing facilities and trees and separating sewage and stormwater systems reflected improvements in Existing buildings and infrastructure (RE02) and Water strategy (RE05). Lastly, for TM, pedestrian-automobile separation and enhanced aesthetics of neighborhood streets supported the positive rating of Safe and appealing streets (TM02). Access to public transport (TM04) and Public transport facilities (TM06) also received “1” because all studied neighborhoods can access at least five bus stations within a 500-meter radius.

In contrast, the other 17 indicators were rated “0”. First, because supplying additional housing, providing resident training and skills, and boosting the local economy were beyond the project missions, the respective indicators (SE01/05/17) were rated “0”. Second, Ecology strategy, Enhancement of ecological value, and Rainwater harvesting (LE01/04/06) were inadequately addressed due to the limited design intention for biodiversity and infrequent adoption of rainwater harvesting. Third, the general absence of a written energy strategy, limited energy efficiency renovations, and minimal design actions to promote cycling (Fig 5-III) led to negative ratings of Resource efficiency, Energy strategy, Sustainable buildings, Low impact materials, Transport carbon emissions, and Cycling network and facilities (RE06/01/04/05/07, TM03/05). Lastly, the absence of a third-party design review process, general lack of LID maintenance, and, in some cases, residents’ alterations of LIDs (Fig 5-III) contributed to negative ratings of all four governance-related indicators.

#### 4.1.2. Residents’ perspective

The residents spoke about three primary areas of satisfaction and three of dissatisfaction. They are noted by green flags of 6 and red flags of 9 BREEAM-C indicators, respectively, in Fig 4. The rationale for flagging each indicator is presented in S1 Table, and representative quotes are in S3 Table. Residents were the most satisfied with runoff mitigation (SE11/13), increased parking spaces (SE12), and general living environment improvements (SE 06/07, TM02). More specifically, sanitary conditions had been improved. New pervious driveways and sidewalks created a more walkable and interactive environment. Impervious surface reduction and vegetated LIDs increased greenery, and the redesign of community spaces improved both their functions and appearance.

Residents’ dissatisfaction was mostly related to landscape and amenity design, vegetation maintenance, and plant appearance and function. First, regarding landscape and amenity design, some criticized the removal of existing trees, vegetation layout, and design flaws of other amenities (e.g., inadequate sun exposure on the benches). Others complained about the “illegible” design language. One resident noted: “*Why they buried the rotting wood in the ground just does not make any sense to me*.*”* All these comments raised concerns for Landscape (SE05), Resource efficiency (RE06), Local vernacular (SE14), Demographic needs and priorities (SE02), Inclusive design (SE15), and the accountability of Design review (GO03) (see specific rationales in S1 Table). Second, residents complained about LID maintenance (GO04) and commented that property management firms (the most typical party responsible for neighborhood maintenance) were often not held accountable and, hence, lacked motivation for LID maintenance. Third, residents expressed an aesthetic preference for trees and flowers over the sedges and grasses often featured in LID design and complained about increased mosquitos and other insects. While residents agreed that the LID plants looked beautiful immediately upon construction, inadequate maintenance contributed to their growing dissatisfaction.

Place attachment, stormwater knowledge, community engagement process, and institutional trust were interconnected factors that affected residents’ satisfaction. First, residents’ attachment to their homes generated strong resistance to landscape alterations, particularly regarding tree removal/replacement. One resident explained: “*I had a tea olive tree that I took care of for years*, *and they took it away…we asked old trees to be returned to us*, *but they never did*.” Second, limited awareness of stormwater problems and LID functionality contributed to institutional distrust and underappreciation of sponge transformation. For example, two residents disbelieved that LIDs could effectively manage stormwater. They further claimed that SCD, compared to other urgent matters such as poverty, was an unwise investment (SE01). Two other residents considered flooding a rare concern in their neighborhoods (although flooding records showed otherwise) and expressed skepticism toward the bureaucratic system, stating that the renewal projects were tools for authorities to benefit themselves financially. One resident, however, mentioned learning about stormwater issues and LID benefits from TV news and construction observation in their neighborhood and was satisfied with the transformation outcomes. Lastly, problems with engagement and scientific knowledge transfer (GO02) can also create distrust. For example, one resident shared an experience communicating with contractors and researchers: *“A so-called sponge city researcher said we were not allowed to grow flowers in this garden…That guy may be full of knowledge*, *but he really knew nothing*. *What does it mean we cannot grow flowers in a garden*?*”* These exchanges show that further engaging the community in understanding their needs and desires and providing environmental education will be critical for building trust in the administration and renewal process.

#### 4.1.3. Experts’ perspective

The experts addressed four primary challenges they had experienced in neighborhood sponge transformation, including benefit tradeoff, maintenance, community trust, and civic engagement (see representative quotes in S4 Table).

First, the severe space shortage in old neighborhoods made it challenging to balance diverse ecosystem needs and residents’ preferences. For example, one expert noted that limited road widths precluded bike lane provision in many neighborhoods. Another mentioned the difficulty of accommodating a rain garden within a very tight public area while fulfilling the residents’ other requests: *“We went back and forth with local residents about our master plan to meet their needs; some wanted more parking*, *others wanted kids’ play areas*. *It is quite challenging to satisfy everyone’s needs*.*”*

Second, post-construction maintenance was repeatedly mentioned as a challenge. As the maintenance responsibility fell mostly on the property management companies the communities hired or sometimes the residents themselves, experts feared that neither had incentives or qualifications to adequately care for LID facilities. One expert noted: “*These are profit-driven companies; there has to be some sort of interest to make them do it*.” Establishing public-private partnerships to diversify funding sources for maintenance and providing adequate training to property management companies remain considerable challenges for local governments.

Third, community trust was another challenge. Experts mentioned using demonstration project tours to alleviate residents’ concerns over life disruptions and LID functionality, which appeared highly effective in encouraging community buy-in. However, to the governments’ surprise, citizens who favored the demonstration projects’ transformed appearances but were rarely affected by stormwater problems started demanding sponge transformation in their neighborhoods. Decisions not to fulfill such requests have generated skepticism over the neighborhood selection process. One expert noted: “*Some residents asked*, *‘Why has neighborhood X been retrofitted while ours hasn’t*? *Is it because some important person lives there*?*’ You understand we operate within a budget*, *right*? *We do want to cater to their requests*, *but not everyone coming to ask for renovations can get it*.” Despite a comprehensive assessment process, which allocated limited budgets to neighborhoods with the highest flooding severity and most inadequate living conditions, the transparency of that process needed improvement.

Finally, the lack of civic engagement was recognized as another challenge. Experts mentioned low participation rates in exploratory engagement efforts, including pre-and post-construction surveys, design reviews, and community meetings. One expert noted: “*Only a small number of residents were interested in joining community meetings*. *They were often community leaders who felt empowered to speak up and influence the sponge construction*.” Moreover, experts showed varying degrees of understanding of community engagement and a general hesitation in investing in engagement due to the cost-benefit uncertainty. For example, one commented: *“Sponge city development is a government-led program…it is difficult for the public to understand the sponge city concept and specific functions in-depth…most people do not need to know all those details*.*”* Another showed concern about the *actual* payback of engagement and potential risks in delaying project completion. Therefore, issues from both sides contributed to the limited influence of residents on project outcomes.

Regarding the experts’ BREEAM-C ratings (see S1 Table), 16 indicators received an aggregated score of “1”, indicating satisfactory performance (Fig 4). They included flood mitigation (SE03/13), public space, facility and service provision (SE06/07/08/11/12/14, LE05, TM02), water management and ecology (LE01/03/04/06, RE03), and consultation and engagement (GO01/02). Conversely, 24 indicators received “0”, among which 12 were deemed “approaching satisfactory performance” and the other 12 “poor performance.”

### 4.2. Policy assessment

The prevalence of BREEAM-C indicators in the national and provincial OUNR policies revealed both policy emphases and gaps (Fig 4). It should be noted that the prevalence values were not directly comparable between the two levels of policies due to substantially different policy document lengths (Table 3).

Regarding the national policy, the category prevalence ranking (i.e., SE > GO > LE > RE > TM) (Fig 4) showed that Social and economic well-being (SE, 11.59%) and Governance (GO, 10.48%) were the most emphasized, while Transport and movement (TM, 0%) was unmentioned. Regarding individual indicator prevalence, 17 indicators were not mentioned in the national policy, including 4 (out of 6) LE indicators (LE01/03/06/04), 7 (out of 17) SE indicators (SE13/11/03/04/16/17/05), and all six TM indicators. In contrast, Land use (LE02, 5.86%), all four governance-related indicators (2.62–4.69%), Delivery of services, facilities, and amenities, Demographic needs and priorities, and Utilities (SE07/06/09, 2.55–2.98%) were the most mentioned. More specifically, first, the functional optimization of shared structures and spaces (LE02) was emphasized. Local administrations were encouraged to renovate underused, vacant, or illegally occupied structures and outdoor spaces into multi-functional community infrastructure. Second, considerable attention was given to participatory governance (GO01-04), wherein residents’ willingness to retrofit and community participation were recognized as essential to OUNR’s long-term success. Third, the focus on Delivery of services, facilities, and amenities, Demographic needs and priorities, and Utilities (SE07/06/09) was manifested in regulations and recommendations of three levels of improvement, i.e., fundamental infrastructure improvement, facilities and services enhancement for all age groups, and upgrades to smart management systems for safety and efficiency.

As for the provincial policies, the category prevalence ranking (i.e., SE > RE > GO > LE > TM) was similar to that of the national. SE (22.18%) and TM (1.70%) remained the top and bottom categories. Regarding individual indicators, Utilities (SE09, 8.98%), Delivery of services, facilities, and amenities (SE06, 4.17%), and Community management of facilities (GO04, 3.76%) were the top three emphases. Their specific requirements mostly echoed the national policy. Conversely, six indicators were not mentioned, including Enhancement of ecological value (LE04), Economic impact (SE01), Training and skills (SE17), Housing provision (SE05), Access to public transport (TM04), and Public transport facilities (TM06).

The national and provincial policies differed in the presence of 14 indicators. Specifically, one indicator (SE01–Economic impact) was only mentioned in the national policy, whereas 13 others were only addressed in provincial policies. They included Ecology strategy (LE01), specific water strategies (LE03/06/RE03/SE13/11/03), noise and light pollution (SE04/16), and streets and cycling-related considerations (TM01/02/03/05). Whereas the national policy generally advocated for a *green* renewal, implicitly targeting broad environmental objectives, the provincial policies provided specific guidelines for those 13 indicators. Lastly, five indicators were absent from either type of policy, including Enhancement of ecological value (LE04), Training and skills (SE17), Housing provision (SE05), and public transport considerations (TM04/06).

### 4.3. Collation of project and policy assessments

**Fig 6:**
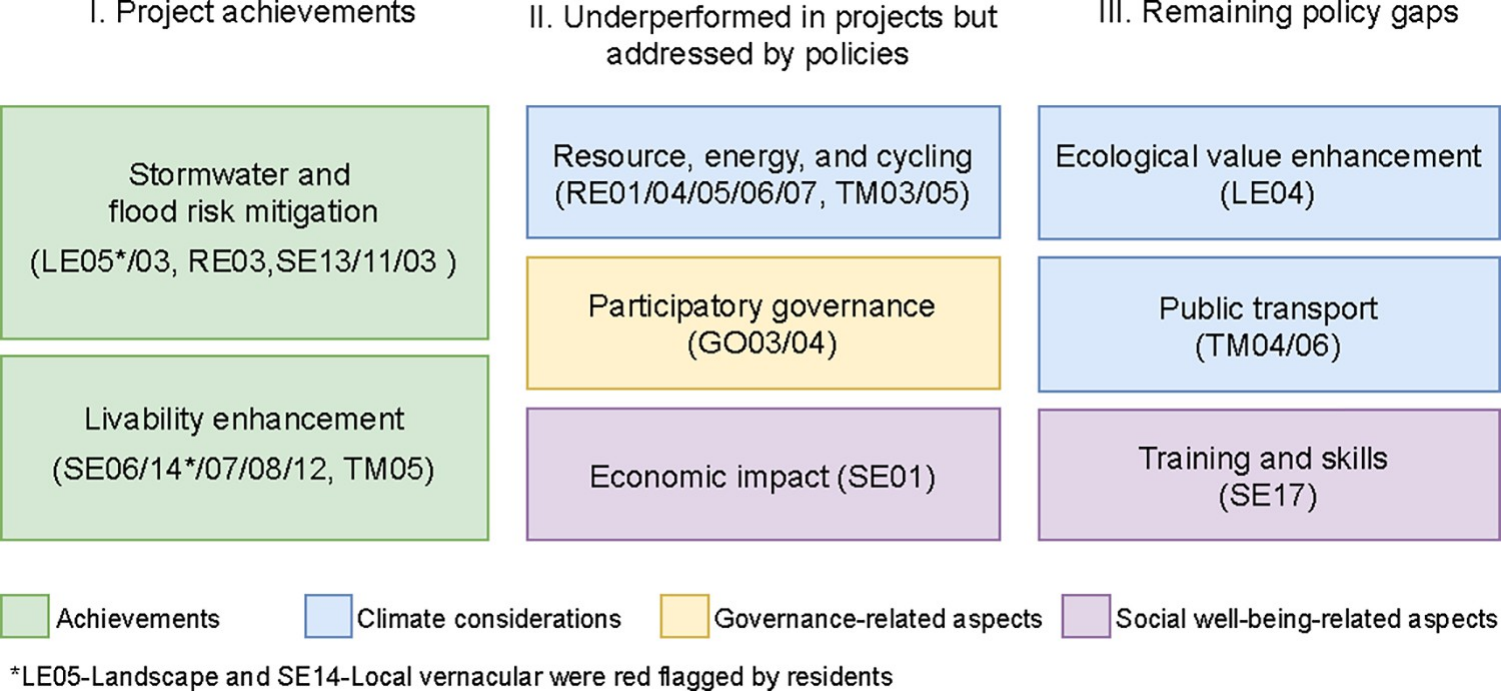
Summary of project and policy assessments.

Upon comparing the project and policy assessments, we summarized the project successes, underperformed areas in projects but addressed by policies, and remaining policy gaps (Fig 6). We focused on the indicators with an aggregate score of “2” (12 indicators) or “0” (13 indicators) to tease out project successes and failures (Fig 4). The former indicates generally favorable outcomes for the indicators of Landscape, Water pollution (LE05/03), Water strategy (RE03), Delivery of services, facilities, and amenities, Local vernacular, Public realm, Microclimate, Flood risk management and assessment, Local parking (SE06/14/07/08/13/11/03/12), and Safe and appealing streets (TM02), although residents raised red flags for Landscape and Local vernacular (LE05/SE14). In contrast, the other 13 indicators scored as “0” indicate underperformed areas, including Design review and Community management of facilities (GO03/04), Enhancement of ecological value (LE04), Energy strategy, Resource efficiency, Sustainable buildings, Low impact materials, Transport carbon emission (RE01/04/05/06/07), Economic impact, Housing provision, Training and skills (SE01/05/17), and Cycling facilities and network (TM03/05) (Fig 4).

Three of the 13 underperformed areas, i.e., Enhancement of ecological value (LE04), Training and skills, and Housing provision (SE05/17), in addition to Access to public transport and Public transport facilities (TM04/06), were not addressed by either type of policy. It should be noted that Housing provision is excluded from subsequent discussions due to the existing high density of these neighborhoods.

## 5. Discussion

The analytical framework presented has filled several essential gaps in the neighborhood sustainability literature. First, our interdisciplinary approach of combining biophysical and technical evaluations with resident and expert perspectives has contributed to a holistic understanding of the transformations brought by the OUNR/SCD initiatives. Second, although experimental policymaking has a longstanding history in China, detailed analyses of the impact of local experiments on regional and national policy initiatives are critically needed. For the section below, as per RQ1 and RQ2, we discuss the sustainable development outcomes and broader implications of the project and policy experiments.

### 5.1. Sustainable development outcomes

The pilot projects’ primary achievements lie in the enhanced hydrological functions, mitigated environmental hazards, and enhanced livability, as reflected by the favorable assessments for 12 specific sustainability indicators (Fig 6-I). The stormwater goals set forth by the central government ensured that flood risk mitigation and LID implementation received top priority. Additionally, the policy goal to improve quality of life resulted in visible and immediate improvements to streets, parking, shared green space, amenities, services, and sometimes housing and utilities. Compared to the large-scale old urban neighborhood demolition and reconstruction from Phases 2 and 3, these Phase 4 projects contributed to curbing urban sprawl and maintaining neighborhood continuity. Their regenerative orientation thus avoided the most negative consequences of the previous approach, including ecosystem destruction, gentrification, and population and cultural displacement [67].

The project and policy experiments underperformed, however, for climate considerations (LE04, RE01/04/05/06/07, TM04/06), participatory governance (GO03/04), in addition to two social-economic well-being indicators, Economic impact and Training and skills (SE01/17) (Fig 6). In what follows, we detail the shortcomings and implications for the two critical areas of climate considerations and participatory governance while briefly discussing the others.

### 5.2. Climate considerations

Climate-related indicators, i.e., resource and energy, cycling, ecological enhancement, and public transport (Fig 6 Panels II and III), received low attention in either project or policy experimentation, reflecting inadequate considerations on how OUNR could respond to climate change.

Although both national and provincial OUNR policies integrated resource and energy guidelines, it will be vital for governments to escalate their actions, as OUNR could play a critical role in reducing building energy demand [68] and meeting China’s climate commitment [22]. However, only one of the four cities–Zhenjiang–leveraged parallel energy efficiency programs to implement building retrofits such as pitched roofs, insulated windows, and energy-efficient lightbulbs [69]. Broadening such efforts will not only help tackle the resource and energy challenges but also generate cost savings.

Moreover, a lifecycle-orientated approach to designing, constructing, and managing the entire neighborhood environment is critical. For example, regarding the landscape, LID construction should preserve mature trees to improve quality of life, stormwater management, and carbon sequestration. Native vegetation that requires minimal long-term maintenance should be promoted to preserve native biodiversity and enhance climate resilience, while supplementing ornamental planting may be necessary to accommodate community preferences.

Next, the projects’ underperformance in cycling and insufficient policy attention to transport and movement, in general, is unfortunate. Although the high density within and surrounding the neighborhoods played a role, the marginalization of bicycle use was also due to the absence of national or local cycling strategies and policies, city forms that favored cars, and the cultural association of car use with affluence. Considering the generally advanced public transportation systems in China’s old city areas, priority should be given to refining national policies to promote infrastructure projects and social programs that enhance bicycle safety and convenience and encourage transport mode changes [70].

To be sure, integrating all of these diverse environmental and social needs into comprehensive urban policies is a significant challenge. Because local discretion in planning and redevelopment projects does not necessarily serve global environmental goals [71], provincial and national-level policies are likely necessary to develop specific action plans embedded within clear organizational structures. Ultimately, China’s march to the net-zero emissions goal by 2060 will require every urban sector to leverage its top-down governance structure to adapt to climate change.

### 5.3. Participatory governance

Another significant limitation of the pilot projects is inadequate civic engagement, both in discussions and decision-making. The consequences of disengagement were evident in resident interviewees’ resistance and skepticism. Residents were dissatisfied, for example, with several aspects of amenity and landscape design, whereas experts rated satisfactory performances on them. While the pilot projects did attempt to consult residents, local authorities remained in the driver’s seat for project decisions [72]. While it is evident that the latest OUNR policies exhibited a strong intention to prioritize public participation, formal processes and enforcement mechanisms are likely necessary to ensure that residents’ priorities are reflected in decision-making at all project stages.

This will require two-way knowledge transfer and science communication: seeking residents’ input on local conditions, working with residents to set priorities and identify solutions, and providing residents with accessible information to help them understand the relevant environmental issues and options. Mandating public hearings before final design decisions and integrating residents’ post-occupancy evaluations into local government performance evaluations may also help empower residents in decision-making. Additionally, providing citizen science training (e.g., audit tools and maintenance skills) can encourage residents to take pride in post-construction stewardship and help ensure the projects’ long-term success [73].

A key barrier to establishing these engagement procedures is the paucity of participatory governance experience among technocrats as they operate under tight project timelines. Collaborative research is greatly needed to identify engagement methods suitable for contemporary Chinese contexts. Officials and professionals will need training in not only the skills but also the value of public participation so that they will enter upcoming experiments with best practices and confidence.

### 5.4. Broader implications

Global cities emerge from distinct political-economic contexts and are at varying phases of urban regeneration, making it challenging to directly compare sustainable development approaches and outcomes. In general, the assessed projects reinforced the unbalanced emphasis on environmental performance over other sustainability criteria, but showed distinct objectives and outcomes for social, economic, and institutional sustainability from many Western studies.

First, the pilot projects’ water management priority follows the trend in many developed countries where water and energy are the central motives of urban environmental sustainability (e.g., US’s Low Impact Development, Germany’s Energy Concept) [74, 75]. Because only a fraction of the pilot projects integrated energy programs, how the OUNR programs progress in their energy performance remains to be assessed by future studies. However, it should be noted that there has been a general lack of quantitative evidence worldwide regarding localized water and/or energy progress. Additionally, a universal underperformance in climate adaptation remains, especially concerning renewable energy use, public transport, nature-based infrastructure, and biodiversity. Critical social, financial, administrative, and knowledge barriers will need to be overcome to enhance environmental performance in these aspects [76].

Next, regarding social sustainability, concerns on the OUNR projects have been mostly about limited considerations of local needs due to inadequate civic engagement. This differs from the primary social equity and livability concerns, such as gentrification, frequently cited in Western literature [4, 45, 46]. Here, while the compound-only regeneration approach does not displace any apartment owners, follow-up studies should investigate its possible long-term effects on property value and low-income renters. Meanwhile, analyses of the spatial distribution and investments of OUNR projects at the city scale are necessary to assess the equity in resource allocation.

Third, the pilot projects’ lack of economic goals reflects a shared challenge of publicly funded regeneration projects [44], where social goals took precedence over monetary ones. However, as countries with a longer regeneration tradition (e.g., the UK) emphasize, strengthening the local economy is one of the most significant components of creating sustainable communities. The ultimate goal is to achieve environmental, social, economic, and institutional coherence [5]. Therefore, identifying a pathway forward to break the residence boundaries and embed neighborhood regeneration into the broader urban development framework will be critical to helping the OUNR program bring essential economic benefits to the area [36].

Fourth, concerning institutional sustainability, China’s top-down governance and experiment-oriented policy process can produce both efficiency and uncertainty in environmental practices and outcomes. In the worst-case scenario, when top-down decisions are passed down with passive obeyance, experimentation can be driven by imported policy formulas that ignore local circumstances. The participatory governance discussions above can help avoid such negative tendencies. In the best-case scenario, however, a top-down process provides critical financial resources and ensures consistency and efficiency in decision-making across multiple government levels. Under China’s current land policies, whereby no real estate property tax is being collected, local governments receive minimal *direct* financial gain from the projects themselves. Given the limited incentives and mechanisms for the market, local governments, or grassroots communities to implement OUNR projects, the SCD experimentation effectively acted as a regeneration catalyst without displacing residents or demanding any capital costs out of residents’ pockets. Although long-term financial challenges will emerge, the incentive structure that jumpstarted such reforms provides inspiration for neighborhoods elsewhere with similar limited incentives and resources.

Finally, utilizing the BREEAM-C framework in a Chinese context proved effective in revealing areas of success and underachievements. In particular, integrating biophysical and technical evaluations with resident and expert perspectives generated a comprehensive understanding of the transformation outcomes. While developing plausible neighborhood sustainability assessment tools for Chinese cities is beyond the study scope, we offer several thoughts on BREEAM-C’s applicability and potential enhancements. Generally, BREEAM-C’s comprehensive criteria encompassing environmental, social, economic, cultural, and governance aspects and simplicity of use provide great promise for its application in China. Its adoption can also facilitate collaboration and knowledge exchange between practitioners in China and their global counterparts, contributing to a shared understanding of sustainable practices and innovations. However, BREEAM-C’s specifications show strong linkages to its country of origin (i.e., the UK) concerning development scale and form, regulations, standards, way of living, and culture. For example, regulatory and procedural-related indicators, such as design review and community engagement, may not align with Chinese regulatory frameworks and standards and, therefore, need to be adapted for the distinct governance structure and process in China’s neighborhood renewal projects. The criteria’s rigid nature may also pose challenges in accommodating the diverse variations in climate, development scale and form, and cultural practices across different cities in China. Moreover, BREEAM-C has been criticized for being static, only able to assess a single outcome rather than the progress of a long-term multi-phase project [55]. Additionally, the comprehensive, up-to-date data required for an accurate assessment may be challenging to obtain, considering Chinese cities’ rapid (re)development speed and general lack of monitoring efforts. Finally, for the current old urban neighborhood regeneration initiative in particular, certain indicators, such as land use and housing provision, do not apply to the compound-only regeneration approach. Others, including those related to larger-scale public transportation improvement, have limited applicability.

## 6. Conclusions

Through a mixed-methods approach consisting of site investigations, expert and resident interviews, and policy document analysis, we examined the sustainability performances of pilot projects under the OUNR and SCD initiatives in China, recent neighborhood regeneration policy emphases and gaps, as well as broader implications of the project and policy experiments. We found that top-down technocratic management here, as implemented through pilot projects, was highly efficient in enhancing hydrological performance and improving the physical environment (e.g., streets, parking, amenities, and services). At the same time, these successes were undermined by the lack of emphasis on climate considerations (e.g., resource and energy efficiency and cycling programs) and participatory governance (e.g., resident inclusion in decision-making). Additionally, although the latest neighborhood renewal policies address most of the sustainability indicators assessed in this study, stronger implementation procedures and enforcement mechanisms are still needed.

The study’s major contributions lie in its timely and comprehensive guidance for China’s expansive urban regeneration endeavors in both sustainable practices and policymaking. The ongoing, large-scale implementation of neighborhood regeneration, a cornerstone of urban renewal in China, underscores the immediate relevance of our research. By addressing the significant literature gap in pilot project assessments, our study provides essential insights to shape the trajectory of future neighborhood regeneration practices. Furthermore, situating the study findings within an international context extends the impact beyond national borders. This broader perspective fosters a global conversation about effective urban renewal practices under contrasting governance structures and cultural contexts, creating a platform for mutual understanding, knowledge exchange, and collaboration on sustainable urban development strategies. Last but not least, our research delves into the applicability of BREEAM-C within the Chinese OUNR context, offering insights into its advantages and limitations. Beyond the enrichment of BREEAM-C-related literature, these insights help pave the way for the future development of neighborhood sustainability assessment tools tailored to the intricacies of China’s urban renewal processes.

Creating people-centered, economically vibrant, and environmentally sustainable neighborhoods is a common goal for global cities with different socio-political structures. No single regenerative approach universally applies. As China continues its sustainable urban development pursuit, civic engagement, local sensibilities, science-informed decision-making, adequate expertise on professional teams, and regular performance assessment are all critical for avoiding the negative tendencies of a primarily top-down approach. What the OUNR and SCD initiatives can offer as inspirations to the broader world are the much-needed state funding to kickstart the reform without resident displacement, potential decisiveness that the top-down approach carries, and the adaptive capacity built by the project and policy experimentations. By trying out locally derived solutions and making incremental changes, best practices can be selectively adopted throughout the system. Assessments such as ours provide critical insights into performance and significantly contribute to advancing urban neighborhood sustainability.

## Supporting information

S1 Table Explanations of BREEAM indicators and rationale for site, resident, and expert assessments. (PDF)

S2 Table Types of LID adoption in pilot neighborhoods. (PDF)

S3 Table Residents’ perspectives. (PDF)

S4 Table Experts’ perspectives. (PDF)
