# Supplementary material for: Sustainability outcomes and policy implications: Evaluating China’s “old urban neighborhood renewal” experiment
Source: PLoS One. 2024 Apr 30;19(4):e0301380. doi: 10.1371/journal.pone.0301380 (PMC11060563; doi:10.1371/journal.pone.0301380)
Supplement: S1 Table — (PDF) [file pone.0301380.s001.pdf]

Supporting Information
Sustainability outcomes and policy implications: Evaluating China's "old urban neighborhood renewal" experiment

S1 Table. Explanations of BREEAM indicators and rationale for site, resident, and expert assessments.

Note:

SI = Site investigation; NA = not assessed due to inadequate information.
RI = Resident interview; G = green flag; R = red flag; NM = not mentioned by residents.
ES = Expert survey. The mean expert rating for each indicator is provided in the table. Note that each expert rated the level of achievement for each indicator based on the following scale: I = irrelevant (0), II = not considered (1), III = poor performance (2), IV = approaching satisfactory performance (3), V = satisfactory performance (4), and VI = strong performance (5). Mean values > 4 indicate overall satisfactory performance; those ranged 3~4 "approaching satisfactory performance," and those < 3 "poor performance."

Indicator	Aim	Rationale for site/resident/expert assessments		Assessment
**Category: GO - Governance**<br>**Definition:** Promotes community involvement in decisions affecting the design, construction, operation, and long-term stewardship of the development.				
GO 04 - Community management of facilities	To support communities in active involvement in developing, managing and/or owning selected facilities.	SI	We observed that residents randomly parked bikes and vehicles in shared open spaces (not designated for parking) in 4 neighborhoods; behavioral issues, such as altering LIDs for personal uses or placing personal items into the planters, were present in 4 neighborhoods; issues of littering, bare planters, and accumulated sediments were observed in 3 neighborhoods. Secondary materials showed complaints on local online forums about the construction quality and maintenance:<br>*http://appshare.chizhouren.com/wap/thread/view-thread/tid/1111565*<br>*https://js.qq.com/a/20180727/016475.htm*<br>*https://jiaxing.19lou.com/forum-778-thread-102711526860998498-1-1.html*	0
		RI	Three residents were dissatisfied with LID maintenance, and they mentioned the lack of maintenance from property management companies.	R
		ES	3.7 (approaching)	0

Indicator	Aim	Rationale for site/resident/expert assessments		Assessment
GO 02 - Consultation and engagement	To ensure the needs, ideas, and knowledge of the community and key stakeholders are used to improve the quality and acceptability of the development throughout the design process.	SI	As shown in GO04, in some cases, residents altered LID facilities for personal usages, such as vegetable/flower gardens, seating areas, and chicken coops. This indicated that residents' functional and cultural needs were not met adequately. Additionally, educational programs as part of the outreach process were not available or effective to enable residents to value and therefore protect LID facilities.	0
		RI	Unpleasant engagement experiences were brought up by residents. One resident had a misunderstanding about LID functions, and their family argued with a sponge expert about how they should use the rain garden in a "correct" way. This manifested that the "different languages" spoken by experts and residents could contribute to social friction and tensions. Another resident claimed that they supported neighborhood transformation but held that they could not change how the government makes decisions.	R
		ES	4.2 (satisfactory)	1
GO 01 - Consultation plan	To ensure the needs, ideas, and knowledge of the community are used to improve the quality of stakeholder engagement throughout the design, planning, and construction process.	SI	Secondary information showed that consultation events such as design reviews and community meetings occurred in four cities, but no formal consultation plans were available.	0
		RI	One resident mentioned that a research team came to their neighborhood and distributed some questionnaires. However, he/she did not know if the team was part of local authorities to collect feedback or independent research institutions for academic research purposes.	NM
		ES	4.2 (satisfactory)	1
GO 03 - Design review	To ensure that the master plan's design is reviewed by the community and other key stakeholders, ensuring that it supports a vibrant, healthy,	SI	A third-party design review process was absent according to secondary information; additionally, the online forum complaints and maintenance issues mentioned above supported the inadequate considerations of local knowledge and preference.	0
		RI	Residents made various complaints about specific design components of sponge transformation.	R

Indicator	Aim	Rationale for site/resident/expert assessments		Assessment
	functional, and inclusive development.	ES	3.8 (approaching)	0
**Category: LE - Land use and ecology**<br>**Definition:** Encourages sustainable land use and ecological enhancement.				
LE 02 - Land use	To encourage the use of previously developed or contaminated land and avoid land that has not been previously disturbed.	SI	The regenerative approach does not disturb new land. LIDs were implemented in vacant or existing green spaces. Some informal structures were demolished, and the reclaimed space was repurposed as new facilities/amenities.	1
		RI	Not mentioned	NM
		ES	3.8 (approaching)	0
LE 05 - Landscape	To ensure that the character of the landscape is respected and, where possible, enhanced through the location of features and design appropriate to the local environment.	SI	Significant improvements in neighborhood tidiness and appearance of shared outdoor space based on comparing pre- and post-construction photos.	1
		RI	Some residents criticized the removal of existing trees, the way vegetation was laid out relative to the paved area, and the design flaws of other amenities (e.g., the lack of sun exposure on the benches and vaguely purposed metal structures that were not useful for any particular age group). Others complained about the "illegible" design language.	R
		ES	4.2 (satisfactory)	1
LE 01 - Ecology strategy	To ensure that the development protects existing natural habitats wherever possible and practical and where not, minimizes and mitigates its impact on existing habitats and promotes measures to enhance biodiversity on-site and in the locality.	SI	Ecological design strategies were mostly only LID-related. Other measures that enhance biodiversity, such as the adoption of a diverse native plant palette, were uncommon.	0
		RI	Not mentioned	NM
		ES	4.0 (satisfactory)	1

Indicator	Aim	Rationale for site/resident/expert assessments		Assessment
LE 03 - Water pollution	To ensure that measures are put in place to protect the local watercourse from pollution and other environmental damage.	SI	The application of LID facilities was helpful for capturing runoff pollutants. The separated stormwater and sewer systems also prevented combined sewage overflows during heavy storm events.	1
		RI	Only residents in Jiaxing mentioned drinking water pollution in the region, which was beyond the scope of OUN stormwater retrofitting.	NM
		ES	4.3 (satisfactory)	1
LE 06 - Rainwater harvesting	To ensure that surface water runoff space is used effectively to minimize water demand.	SI	Rain barrels were not commonly applied in neighborhoods. Only one residence installed rain barrels. Secondary material showed that another neighborhood had an underground stormwater cistern to collect runoff for landscape irrigation.	0
		RI	Not mentioned	NM
		ES	4.3 (satisfactory)	1
LE 04 - Enhancement of ecological value	To ensure that the ecological value of the development is maximized through enhancement.	SI	All studied neighborhoods were in highly urbanized areas with little existing natural habitat in or surrounding the neighborhoods. Secondary material indicated minimal considerations in maximizing the ecological value of the neighborhoods. Native plant diversity was not universally emphasized in the neighborhood redesign, although we observed on-site that two residences implemented low-maintenance native planting designs.	0
		RI	Not mentioned	NM
		ES	3.8 (approaching)	0
**Category: RE - Resource and energy**<br>**Definition:** Addresses the sustainable use of natural resources and the reduction of carbon emissions.				
RE 02 - Existing buildings and infrastructure	To take account of the embodied carbon in existing buildings and infrastructure and to promote their reuse where possible.	SI	The sponge transformation preserved neighborhoods' existing buildings for continuous use. Two neighborhood building façades were renovated, and all neighborhoods' drainage systems were updated.	1
		RI	Not mentioned	NM
		ES	3.4 (approaching)	0

Indicator	Aim	Rationale for site/resident/expert assessments		Assessment
RE 06 - Resource efficiency	To promote resource efficiency by reducing waste during construction and throughout the life cycle of the development.	SI	Except for Zhenjiang, which has integrated a parallel energy efficiency program into sponge transformation, no other neighborhoods formally adopted plans to address resource efficiency.	0
		RI	Two residents complained that some landscaping strategies were a waste of resources. For example, wood pieces were placed in the ground with few practical or aesthetic functions from their perspectives. The replaced soil in the LIDs appeared poor in nutrients and, therefore, did not support the plants well. Three residents thought investing money in sponge transformation was a waste of money.	R
		ES	3.2 (approaching)	0
RE 01 - Energy strategy	To recognize and encourage developments designed to minimize operational energy demand, consumption, and carbon dioxide emissions.	SI	No formal plans have been found addressing the energy consumption reduction in the neighborhoods. As mentioned above, only Zhenjiang integrated the energy efficiency program with sponge transformation by adopting pitched rooftops, energy-saving lightbulbs, and renovating building envelopes.	0
		RI	Not mentioned	NM
		ES	2.9 (poor)	0
RE 04 - Sustainable buildings	To increase the sustainability of all buildings within the development.	SI	Only Zhenjiang retrofitted buildings with sustainable building standards. (*http://news.jstv.com/a/20170919/1505785903184.shtml*). The pilot sponge transformation period had not seen a widespread convergence with other programs (e.g., energy efficiency program) yet partly due to agency silos. Only when a neighborhood was simultaneously enlisted by multiple programs could that neighborhood have the two renovations integrated.	0
		RI	Not mentioned	NM
		ES	3.3 (approaching)	0

Indicator	Aim	Rationale for site/resident/expert assessments		Assessment
RE 03 - Water strategy	To ensure that the development is designed to minimize water demand through efficiency and appropriate supply-side options, taking full account of current and predicted future availability of water in the area.	SI	All the neighborhoods had their stormwater systems renovated. Downspouts were disconnected with the municipal combined/stormwater system, allowing rooftop runoff to be stored/infiltrated/cleansed before being discharged into pipes. Other issues were also corrected to reduce leaking, water pollution, and unauthorized water connection with the municipal sewer system. *(https://k.sina.cn/article_1829407315_6d0a8a53020003m5b.html?from=news&subch=onews. https://z.hangzhou.com.cn/2017/hzhmcs/2020-06/03/7e442824-497c-4d19-92da-e4d462bc5066.pdf)*	1
		RI	Not mentioned	NM
		ES	4.2 (satisfactory)	1
RE 05 - Low impact materials	To reduce the environmental impact of construction through the use of low-impact materials in the public realm.	SI	Low-impact materials were not commonly applied in all neighborhoods, although some experiments have been conducted, such as applying locally reclaimed materials according to secondary information (*https://www.sohu.com/a/339483164_114731*).	0
		RI	Not mentioned	NM
		ES	3.7 (approaching)	0
RE 07 - Transport carbon emissions	To reduce pollution associated with car use and provide viable alternatives to car ownership.	SI	Although several neighborhoods had bike garages, bike lanes were uncommon. No cycling programs from the cities have been found. Residents' requests for more parking spaces were prioritized, which undermined the provision of transportation alternatives.	0
		RI	Not mentioned	NM
		ES	1.3 (poor)	0

Category: SE - Social and economic well-being<br>Definition: (1) Local economy: To create a healthy economy (employment opportunities and thriving business).<br>(2) Social well-being: To ensure a socially cohesive community.<br>(3) Environmental conditions: To minimize the impacts of environmental conditions on the health and well-being of occupants.				
SE 09 - Utilities	To provide easy access to site service and communications infrastructure, with minimal disruption and need for reconstruction, and to allow for future growth in services.	SI	Water-related utilities have been improved in all the neighborhoods. In addition, new waste recycling facilities were provided in over half of the neighborhoods.	1
		RI	Not mentioned	NM
		ES	3.9 (approaching)	0
SE 06 - Delivery of services, facilities, and amenities	To ensure essential facilities are provided and that they are located within a reasonable and safe walking distance.	SI	Frequent new installations of LIDs, pavilions, playgrounds, outdoor seating and lighting, fitness equipment, and street signs were present. In addition, because the studied neighborhoods were located in city cores, they could easily access essential facilities, such as schools, hospitals, and grocery stores.	1
		RI	Residents were satisfied with the increase in parking spaces and general improvements in the living environment. The redesign of shared community spaces improved both their functions and appearance.	G
		ES	4.2 (satisfactory)	1
SE 14 - Local vernacular	To ensure that the development relates to the local character whilst reinforcing its own identity.	SI	The retrofitting approach avoided population and cultural displacement; building and landscape styles were largely consistent with pre-renewal conditions; in some cases, locally sourced materials were applied to street reconstruction.	1
		RI	Several residents mentioned they did not like the appearance of and bug problems associated with new plants and felt upset about losing previous trees.	R
		ES	4.1 (satisfactory)	1

SE 07 - Public realm	To encourage social interaction by creating comfortable and vibrant spaces in the public realm.	SI	Recreational and fitness facilities, benches, and pavilions were commonly provided to create vibrant community spaces and encourage social interactions.	1
		RI	Most residents agreed that sponge transformation improved the living environment.	G
		ES	4.3 (satisfactory)	1
SE 02 - Demographic needs and priorities	To ensure that the development plans for the provision of housing, services, facilities, and amenities are based upon the local demographic trends and priorities.	SI	Accessible facilities were installed for people with disabilities; benches, informational signs, and playgrounds were installed in neighborhoods with central open spaces.	1
		RI	One senior resident mentioned that the installed metal structure was not useful for any particular age group.	R
		ES	2.9 (poor)	0
SE 15 - Inclusive design	To create an inclusive community by enhancing accessibility for as many current and future residents as possible.	SI	Street reconstruction ensured a safer walking environment for all age groups. Accessible facilities, recreational facilities, and other amenities targeting a healthy lifestyle, such as jogging tracks, were observed in neighborhoods with adequate public spaces.	1
		RI	One senior resident mentioned that the installed metal structure was not useful for any particular age group.	R
		ES	3.9 (approaching)	0
SE 10 - Adapting to climate change	To ensure the development is resilient to the known and predicted impacts of climate change.	SI	LID facilities were installed to mitigate flood risk and reduce water pollution. Certain types of LIDs (e.g., vegetated LIDs) could also alleviate air pollution and the urban heat island effect.	1
		RI	Not mentioned	NM
		ES	3.8 (approaching)	0
SE 08 - Microclimate	To ensure the development provides a comfortable outdoor environment	SI	Many types of LID facilities can increase evapotranspiration and regulate microclimate.	1
		RI	Not mentioned	NM

	through the control of climatic conditions on a micro-scale.	ES	4.3 (satisfactory)	1
SE 13 - Flood risk management	To avoid, reduce, and delay the discharge of rainfall to public sewers and watercourses, thereby minimizing the risk of localized flooding on and off-site, watercourse pollution, and other environmental damage.	SI	As mentioned above, various stormwater management strategies have been adopted in all neighborhoods to address SCD's primary goal of mitigating nuisance flooding.	1
		RI	Most residents were satisfied with reduced stormwater runoff and flooding.	G
		ES	4.6 (satisfactory)	1
SE 11 - Green infrastructure	To ensure access to high-quality space in the natural environment or urban green infrastructure for all.	SI	As mentioned above, various types of LIDs have been adopted in all neighborhoods.	1
		RI	Most residents were satisfied with the hydrological performance of LIDs.	G
		ES	4.0 (satisfactory)	1
SE 03 - Flood risk assessment	To ensure that the development takes account of flood risk and, where it is present, takes appropriate measures to reduce the risk of flooding to the development and the surrounding areas.	SI	Secondary information showed that neighborhoods had flood risk assessments in order to redesign the neighborhood stormwater system. Stormwater volume and pollution treatment capacity also have been calculated to demonstrate how hydrological goals were addressed. In some neighborhoods, stormwater quantity and quality monitoring devices were installed to assess actual performance.	1
		RI	Not mentioned	NM
		ES	4.4 (satisfactory)	1
SE 12 - Local parking	To ensure parking is appropriate for the expected users and well integrated into the development.	SI	New parking spaces were added, and existing ones were retrofitted using permeable materials.	1
		RI	Most residents were satisfied with the added parking spaces.	G
		ES	4.1 (satisfactory)	1

SE 04 - Noise pollution	To ensure that the development is designed to mitigate the impacts of noise. This includes mitigation from existing sources of noise, reducing potential noise conflicts between future site occupants, and protecting nearby noise-sensitive areas from noise sources associated with the new development.	SI	Not assessed due to lack of pre- and post-construction information.	NA
		RI	Not mentioned	NM
		ES	2.2 (poor)	0
SE 16 - Light pollution	To ensure that lighting on site is designed to reduce light pollution.	SI	Not assessed due to lack of pre- and post-construction information.	NA
		RI	Not mentioned	NM
		ES	2.6 (poor)	0
SE 01 - Economic impact	To increase economic well-being by ensuring that the development attracts inward investment, creates jobs and complements and enhances existing economic activity in the local area and surrounding economy	SI	Secondary information suggested that economic benefits were not intentionally pursued by the sponge transformation. Site investigation found limited design/management efforts intended to attract investment or create jobs.	0
		RI	Three residents thought sponge transformation was a waste of money.	R
		ES	2.8 (poor)	0
SE 17 - Training and skills	To ensure that the development contributes to the local area by	SI	Secondary information suggested that providing necessary training and skills to residents and stakeholders was not intentionally pursued by the sponge transformation.	0

	enhancing skills and training opportunities.	RI	Not mentioned	NM
		ES	2.8 (poor)	0
SE 05 - Housing provision	To minimize social inequalities and foster a socially inclusive community by ensuring appropriate housing provision within the development.	SI	Housing provision was not a goal set in pilot programs due to the retrofitting nature and high-density environment.	0
		RI	Not mentioned	NM
		ES	2.2 (poor)	0
**Category: TM - Transport and movement**<br>**Definition:** Addresses the design and provision of transport and movement infrastructure to encourage the use of sustainable modes of transport.				
TM 02 - Safe and appealing streets	To create safe and appealing spaces that encourage human interaction and a positive sense of place.	SI	Green streets were applied to improve aesthetics; sidewalks and driveways were separated, and lighting was provided to increase safety; street signs were provided to improve wayfinding and increase community value.	1
		RI	Residents mentioned that renovations of sidewalks and driveways improved walkability, and the community became tidier and more organized than before.	G
		ES	4.1 (satisfactory)	1
TM 01 - Transport assessment	To ensure transport and movement strategies reduce the impact of the development upon the existing transport infrastructure and improve environmental and social sustainability through transport.	SI	NA	NA
		RI	Not mentioned	NM
		ES	3.6 (approaching)	0

TM 05 - Cycling facilities	To promote cycling by ensuring the adequate provision of cyclist facilities.	SI	Bike lanes remained largely absent. Several neighborhoods provided bike garages, but they were not fully used by residents. In many cases, residents preferred to park their bicycles or motorcycles at the entrances of their residential buildings for convenience.	0
		RI	Not mentioned	NM
		ES	2.2 (poor)	0
TM 03 - Cycling network	To promote cycling as a leisure activity and as an alternative to vehicle use by providing a safe and efficient cycle network.	SI	Cycling networks were not available in all the neighborhoods, partly due to the narrow street widths. Generally, cycling networks have not been promoted in the neighborhoods and surrounding areas, implying a lack of consideration for alternative vehicle use in OUNR.	0
		RI	Not mentioned	NM
		ES	2.3 (poor)	0
TM 04 - Access to public transport	To ensure the availability of frequent and convenient public transport links to fixed public transport nodes (train, bus, tram, or tube) and local centers.	SI	Due to their locations in city core areas, all neighborhoods had at least five bus stations within a 500-meter radius, indicating good access to public transport in small to medium-sized Chinese cities.	1
		RI	Not mentioned	NM
		ES	2.1 (poor)	0
TM 06 - Public transport facilities	To encourage frequent use of public transport throughout the year by providing safe and comfortable transport facilities.	SI	As mentioned above, all neighborhoods had good access to public transport within a 500-meter radius.	1
		RI	Not mentioned	NM
		ES	2.2 (poor)	0
