# Supplementary material for: Sustainability outcomes and policy implications: Evaluating China’s “old urban neighborhood renewal” experiment
Source: PLoS One. 2024 Apr 30;19(4):e0301380. doi: 10.1371/journal.pone.0301380 (PMC11060563; doi:10.1371/journal.pone.0301380)
Supplement: S2 Table — (PDF) [file pone.0301380.s002.pdf]

Supporting Information
Sustainability outcomes and policy implications: Evaluating China's "old urban neighborhood renewal" experiment

S2 Table. Types of LID adoption in pilot neighborhoods.

No.	Neighborhoods	Pervious pavement	Tree trench/ planter	Sunken green space	Rain garden /bioswale	Downspout disconnection	Green roof	Rain barrel	Educational board
**1**	Jueyuanchang residence	✓	✓			✓	✓		✓
**2**	Yanyu residence	✓	✓	✓		✓			
**3**	Funan Garden Phase 1	✓	✓	✓	✓	✓			
**4**	Funan Garden Phase 2	✓	✓	✓	✓	✓			✓
**5**	Funan Garden Phase 3	✓	✓	✓	✓	✓			✓
**6**	Huarun Village	✓	✓	✓	✓				✓
**7**	Chashan residence	✓	✓	✓					
**8**	Runjiang residence	✓	✓	✓					
**9**	Jiangbin Village	✓	✓	✓				✓	
**10**	Huashan Village Phase 1	✓	✓	✓		✓			✓
**11**	Zhiye New Village	✓	✓	✓		✓			✓
**12**	Huijing Garden Phase 1	✓	✓	✓	✓	✓			
**13**	Jindian residence	✓	✓	✓	✓	✓			✓
	**Total count**	13	13	12	6	9	1	1	7
