# Supplementary material for: Sustainability outcomes and policy implications: Evaluating China’s “old urban neighborhood renewal” experiment
Source: PLoS One. 2024 Apr 30;19(4):e0301380. doi: 10.1371/journal.pone.0301380 (PMC11060563; doi:10.1371/journal.pone.0301380)
Supplement: S3 Table — (PDF) [file pone.0301380.s003.pdf]

S3 Table. Residents' perspectives.

Themes	No.	Representative quotes
**Satisfaction**		
Improvement of parking	*1*	*"The renovation is quite good, and it brought us more parking spots, you know, some people previously parked personal cars in green spaces."*
Stormwater mitigation	*2*	*"They changed the road surface perhaps two years ago, and it did work because these are pervious pavers… the neighborhood has become tidier than before, and we have more green cover in the outdoor space."*
Road and general public space improvement	*3*	*"I felt good about the renovations. The roads have improved a lot, and it did not look like this at all before."*
	4	*"I really like how my community looks like now; look, this entire open space has improved a lot and become cleaner."*
**Dissatisfaction**		
Landscape & amenity design	*5*	*"This area should be converted to a paved surface to make it flat and open, and then they should plant some flowers around it to make it look clean and ordered; it would be much better than now."*
	6	*"I do not understand why they put this metal stuff here. It is too high for the kids to play on and not good either for sun-drying clothes."*
	7	*"Why they buried the rotting wood in the ground just does not make any sense to me. It is a total waste."*
	8	*"The bench is not useful; it is too low for me, and you do not have sunlight when sitting on it in winter."*
Vegetation maintenance	*9*	*"Flowers were all dying, and no one came to take care of them."*
	10	*"I asked them to replace the tree, which was almost dying; they saw it and didn't do a thing. They only took care of the parking space because that provides a source of revenue."*
Plant functionality & appearance	*11*	*"In summer, you got bug bites on your legs everywhere because the benches were surrounded by those messy grasses."*
	12	*"These grasses attract so many bugs, and they climb into my neighbor's first-floor storage room."*
	13	*"There used to be a tree with red blooms all over its branches, and it looked so pretty."*
	14	*"There used to be two pomegranate trees, and they bloomed beautifully, and the fruit was delicious as well."*
	15	*"These grasses look unsightly; we had much more beautiful flowers and trees here before the sponge renovation."*

Factors affecting residents' perceptions		
Place attachment	*16*	*"This is like our home, and it needs to be clean, right?"*
	17	*"I am willing to clear the ground like I do for my own home and clean a bit under the bench."*
	18	*"I have a tea olive tree that I took care of for many years, and they took it away and did not pay me. It is not just about money - they have to talk to me in advance in order to take my tree, right?... We asked old trees to be returned to us, but they never did. Instead, they planted those worthless trees."*
Understanding of stormwater issues and LID functionality	*19*	*"I know the government wants to do good things about the environment, but I do not support them promoting sponge city development because it is a waste of money, our money, and money should be used for other national affairs such as education in poor regions."*
	20	*"The sponge construction is useless because our neighborhood was not affected by flooding."*
	21	*"…digging a hole is not sponge city development. This is useless."*
Distrust and engagement process	*22*	*"Roads were renovated again and again. That is how they make money out of it."*
	23	*"…a so-called sponge researcher said we were not allowed to grow flowers in this garden… and my husband got so angry and started arguing with him. He ended up being very rude to us…you know, that guy may be full of knowledge, but he really knew nothing. What does it mean we cannot grow flowers in a garden? What should be grown in a flower garden, then?"*
