# Supplementary material for: Sustainability outcomes and policy implications: Evaluating China’s “old urban neighborhood renewal” experiment
Source: PLoS One. 2024 Apr 30;19(4):e0301380. doi: 10.1371/journal.pone.0301380 (PMC11060563; doi:10.1371/journal.pone.0301380)
Supplement: S4 Table — (PDF) [file pone.0301380.s004.pdf]

S4 Table. Experts' perspectives.

Themes	No.	Representative quotes
Benefit trade-off	*1*	*"It's not practical to change the combined sewer systems into separated stormwater sewer systems for all old neighborhoods due to limitations in budgeting, feasibility, and timeline. Instead, we created surface stormwater systems using rain gardens, grass filters, and downspout disconnections to collect, purify, and reuse stormwater. It's not very costly and works really well in terms of landscape aesthetics and stormwater treatment for those old neighborhoods, and we installed this type of system for 18 old neighborhoods."*
	2	*"We went back and forth with local residents about our master plan to meet their needs; some wanted more parking, others wanted kids' play areas. It is quite challenging to satisfy everyone's needs, and we also needed to reiterate the calculation of stormwater treatment volume to meet the goals."*
	3	*"Renovation is difficult…for some historical districts where there was little space for installing LID facilities. We had to somehow use other spaces to meet the pervious pavement requirement so that the overall quantity goals were met in our region."*
Post-construction maintenance	*4*	*"Public projects could receive special funding from municipalities or other private resources; anyway, it is all well-planned. For old neighborhoods, however, I really do not know how sponge facilities will be maintained."*
	5	*"These are profit-driven companies; there has to be some sort of interest to make them do it."*
	6	*"Government should enact some supervision on neighborhood maintenance or provide some kind of incentives for these management companies."*
Community trust	*7*	*"We had governmental properties retrofitted first, and we invited residents to tour these projects. After the demonstration, we, in fact, had many residents call us requesting their neighborhoods to be retrofitted. We had a green roof on our government building, and residents even asked if they could get the same type for their communities, but you know, there is always the budget issue."*
	8	*"People came to us and were skeptical about the selection of neighborhoods for sponge renovations; some had asked: 'why has x neighborhood been retrofitted, but we have not? Is it because some important person lives there?' You know we have our budget, right? We do want to cater to their request, but it is not like that; everyone comes to ask for renovations, and they can get it."*

Civic engagement	*9*	*"We had two community meetings before construction…we invited resident representatives to our design and planning meeting…only a small number of residents were interested in joining such events. They were often community leaders who felt empowered to speak up and influence the sponge construction."*
	10	*"To be honest, most people are not interested and do not have any opinions on it (SCD)."*
	11	*"A resident did not like the sponge transformation. So we started talking to his/her family to get their support and convince her/him. We can even get different opinions just in one family…you can get an idea of how hard the engagement has been in these neighborhoods."*
	12	*"Sponge city development is a government-led program; we want the public to know where the money was spent…it is difficult for the public to understand the sponge city concept and specific functions in-depth…most people don't need to know all those details."*
	13	*"Due to the developing economic conditions and civic virtue levels in these regions, public participation may even pose a high risk to the overall construction progress."*
